# Cost-effectiveness of umeclidinium/vilanterol combination therapy compared to tiotropium monotherapy among symptomatic patients with chronic obstructive pulmonary disease in the UK

**DOI:** 10.1186/s12962-015-0048-6

**Published:** 2015-12-12

**Authors:** Yogesh Suresh Punekar, Graeme Roberts, Afisi Ismaila, Martin O’Leary

**Affiliations:** Value Evidence and Outcomes, GlaxoSmithKline R&D, Stockley Park, Uxbridge, UB11 1BT UK; Double Helix Consulting, London, W1U 6TQ UK; Value Evidence and Outcomes, GlaxoSmithKline R&D, RTP, Durham, NC 27709-3398 USA; Clinical Epidemiology and Biostatistics, McMaster University, Hamilton, ON L8S 4L8 Canada

**Keywords:** Chronic obstructive pulmonary disease, Cost-effectiveness, Tiotropium, Umeclidinium/vilanterol, H5 National Government Expenditures and Related Policies.

## Abstract

**Background:**

The cost-effectiveness of umeclidinium bromide-vilanterol (UMEC/VI) versus tiotropium monotherapy in the UK was assessed using a UMEC/VI treatment-specific economic model based on a chronic obstructive pulmonary disease (COPD) disease-progression model.

**Methods:**

The model was implemented as a linked-equation model to estimate COPD progression and associated health service costs, and its impact on quality-adjusted life years (QALYs) and survival. Statistical risk equations for clinical endpoints and resource use were derived from the ECLIPSE and TORCH studies, respectively. For the selected timeframe (1–40 years) and probabilistic analysis, model outputs included disaggregated costs, total costs, exacerbations, life-years and QALYs gained, and incremental cost-effectiveness ratios (ICERs).

**Results:**

Random-effects meta-analysis of tiotropium comparator trials estimated treatment effect of UMEC/VI as 92.17 mL (95 % confidence interval: 61.52, 122.82) in forced expiratory volume in 1 s. With this benefit, UMEC/VI resulted in an estimated annual exacerbation reduction of 0.04 exacerbations/patient and 0.36 life years gained compared to tiotropium over patient lifetime. With an additional 0.18 QALYs/patient and an additional lifetime cost of £372/patient at price parity, the incremental cost effectiveness ratio (ICER) of UMEC/VI compared to tiotropium was £2088/QALY. This ICER increased to £17,541/QALY when price of UMEC/VI was increased to that of indacaterol plus tiotropium in separate inhalers. The ICER improved when model duration was reduced from patient lifetime to 1 or 5 years, or when treatment effect was assumed to last for 12 months following treatment initiation.

**Conclusion:**

UMEC/VI can be considered a cost-effective alternative to tiotropium at a certain price.

**Electronic supplementary material:**

The online version of this article (doi:10.1186/s12962-015-0048-6) contains supplementary material, which is available to authorized users.

## Background

Chronic obstructive pulmonary disease (COPD), a common preventable and treatable disease, is characterised by persistent and progressive airflow limitation. It is associated with an increased chronic inflammatory response in the lungs to noxious stimuli [1]. It is ranked by the World Health Organisation (WHO) as the fourth leading cause of death worldwide. In 2011, as estimated by the WHO, there were 2.96 million deaths worldwide attributed to COPD [2, 3]. According to data from the quality and outcomes framework report, the prevalence of diagnosed COPD is 1.6 % (estimated 819,524 people) in the United Kingdom; however, an estimated 3 million people have COPD [4].

Globally, the economic impact of COPD is reflected in the 3.3 % disability-adjusted life years (DALYs) reported in the year 2011 by the WHO [5] and the high percentage of patients (nearly 40 %) who are forced to discontinue work [6]. Early treatment of COPD involves the use of long-acting muscarinic antagonists (LAMAs) such as tiotropium, glycopyrronium, and aclidinium or long-acting β2-agonist (LABA) monotherapy such as formoterol, salmeterol, and indacaterol or combination therapy, as necessary. Co-administration of LAMAs and LABAs is more effective in managing stable COPD than either drug class alone, as studies indicate improved lung function, symptoms, and health status with the former [7–9]. The Global Initiative for Chronic Obstructive Lung Disease (GOLD) guidelines recommend the use of a combination of long-acting bronchodilators with differing mechanisms of action if monotherapy is insufficient to control the symptoms of COPD [1]. A second bronchodilator may be considered in moderate COPD to optimise symptom benefit [1, 10] whilst avoiding the risk of side effects associated with dose escalation of a single bronchodilator [10]. However, combination treatment in separate inhalers may potentially lead to other challenges such as lower adherence/persistence and suboptimal outcomes [11, 12].

Umeclidinium bromide-vilanterol (UMEC/VI) is a new fixed-dose LAMA/LABA combination (Anoro®) indicated as maintenance bronchodilator treatment in patients with COPD. It is available at a delivered dose of 55/22 µg once daily in a novel dry-powder inhaler (Ellipta^®^). In the European Union, it is approved as maintenance bronchodilator treatment to relieve symptoms in adult patients with COPD. The safety and efficacy of UMEC/VI has been established through a clinical development programme enrolling more than 8000 subjects with COPD. Three active-comparator phase 3a studies (DB2113360, DB2113374, ZEP117115) [13, 14] in this programme evaluated the efficacy of UMEC/VI combination therapy compared with tiotropium. These studies have demonstrated that UMEC/VI provides significant improvements in lung function compared to tiotropium which has gained worldwide acceptance as a first-line, once-daily maintenance therapy for patients with COPD [1, 15]. UMEC/VI along with other fixed dual bronchodilator combinations may present a new class of initial maintenance treatments in COPD.

Treatment costs are an important consideration in chronic diseases such as COPD. With the increasing number of LAMAs and LAMA/LABA combination therapies in the market, treatment costs are likely to be a major concern to payers. The objective of this study was to assess the cost-effectiveness of UMEC/VI compared with tiotropium monotherapy from the UK National Health Services (NHS) perspective.

## Methods

### COPD disease-specific model

**Fig. 1:**
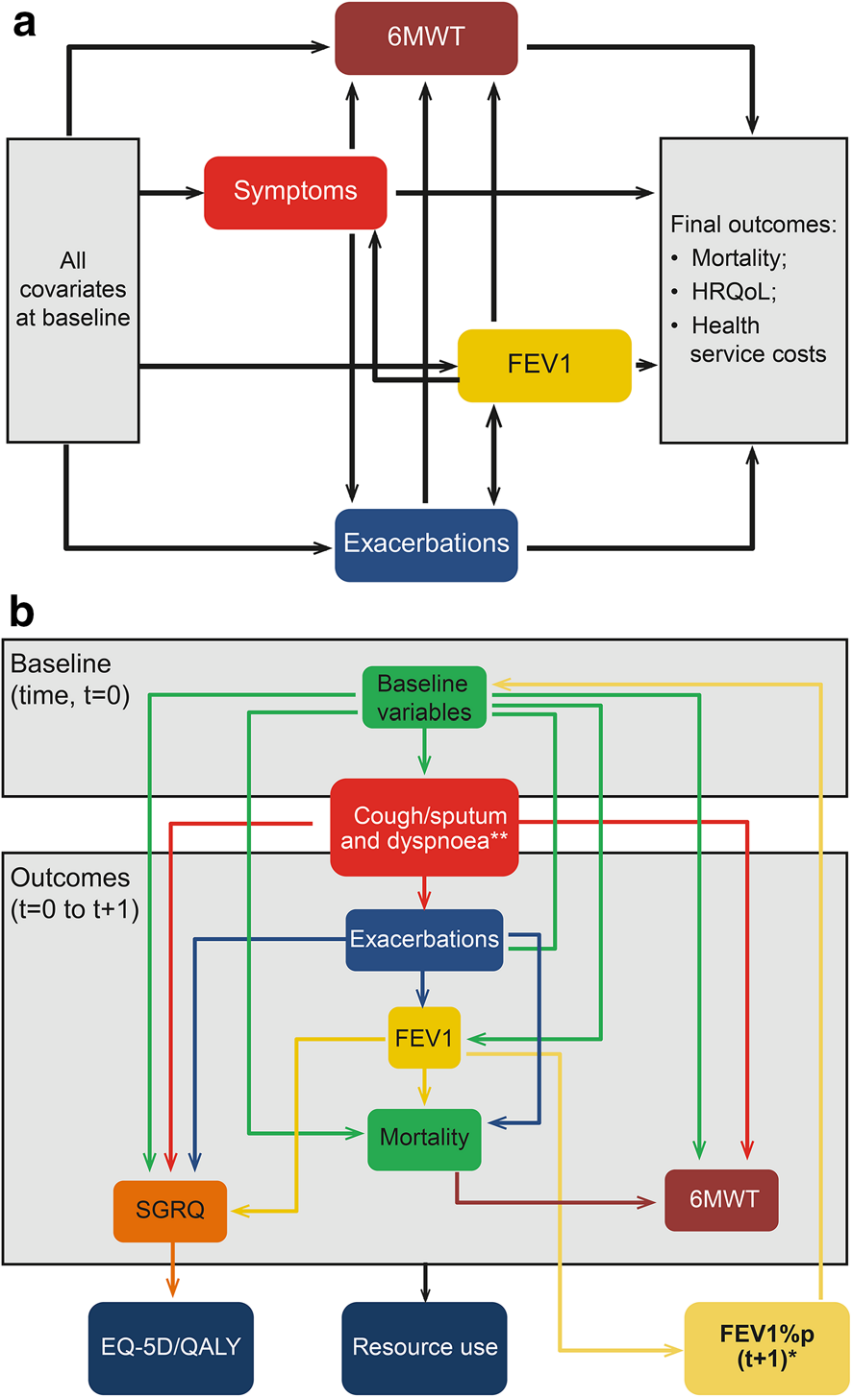
**a** Final conceptual model of COPD. **b** Linked-equations disease progression model for COPD. *6MWT* 6-min walk test, *COPD* chronic obstructive pulmonary disease, *EQ-5D* EuroQol 5 dimension, *FEV1* forced expiratory volume in 1 s, *FEV1%p* forced expiratory volume in 1 s percent predicted, *HRQoL* health-related quality of life, *QALY* quality-adjusted life year, *SGRQ* St. George’s Respiratory Questionnaire. **a** Adopted from Tabberer et al. [15]. **b** Adapted from Briggs et al. [14]. **a** Figure represents association between baseline covariates, intermediate outcomes (FEV1, symptoms, exacerbations and 6MWT) and final outcomes (mortality, HRQoL and costs). *Arrows* represent direction of effect e.g. baseline covariates affect intermediate outcomes. **b** Figure represents schema of linked equations model. Baseline covariates at t = 0 predict intermediate and final outcomes at t = 1 (model cycle = 1). These then become baseline covariates t = 1 to predict intermediate and final outcomes at t = 2 (model cycle = 2)

The economic model used in this evaluation was adapted from a COPD disease model published elsewhere [16]. The disease model itself was based on a conceptual model of disease progression. This conceptual model provided a framework to describe relationships between different demographic and clinical parameters, disease progression, and health outcomes (Fig. 1a) [17]. This association was estimated using data from the Evaluation of COPD Longitudinally to Identify Predictive Surrogate Endpoints (ECLIPSE) study [18] and the resulting risk equations were connected through a model that predicted utility, survival, and health-care resource use in future [16, 19] (Fig. 1b; Additional file 1).

### Model description

Input parameters in the COPD disease model include age, gender distribution, body mass index, cardiovascular and other comorbidities, prior exacerbation history, smoking status and health status measured by the SGRQ or St George’s Respiratory Questionnaire for COPD (SGRQ-C). The model also requires baseline estimates for proportions of patients with dyspnoea, cough and sputum, exacerbation rate, forced expiratory volume in one second (FEV1), and exercise capacity measured by the 6-min walk test (6MWT). One or more of these can either be input directly in the model or predicted using other available parameters [16, 19].

In each model cycle, based on the statistical risk equations (Additional file 1) and by using the baseline clinical and demographic information of the target population, the model estimates the number of moderate and severe exacerbations, predicted FEV1 in millilitres, the proportion of patients with dyspnoea or cough and sputum symptoms most days per week, 6MWT distance in metres, and SGRQ total score (Fig. 1b). These parameter estimates then predict SGRQ scores, survival, and resource use in that model cycle. All of these parameters have been shown to be good predictors of future disease progression in COPD and are therefore used as input parameters in the subsequent cycles [17]. For each time period, the predicted SGRQ scores were transformed to the EQ-5D utility based on a published algorithm [20]. The risk equations providing annual rates were further adjusted to estimate outcomes at specific cycle lengths.

### UMEC/VI treatment-specific economic model

In order to fit this model to UMEC/VI clinical programme, a 6-month trial period was added at the start. It was implemented as two 3-month cycles, and 6-monthly cycles thereafter for the remainder of the model timeframe. In the base case, the model time frame was assumed to be over the lifetime of the patients. Separate scenario analyses were conducted to assess the benefit of UMEC/VI over shorter time frames of 1 year (Scenario A) and 5 years (Scenario B).

#### Model inputs

The baseline cohort used in the model represented the UMEC/VI phase 3a trial population derived using an integrated analysis of four pivotal trials (DB2113360, DB2113361, DB2113373, DB2113374) [13, 21, 22]. In instances where such information was not available from the UMEC/VI clinical programme, baseline estimates from ECLIPSE [18] were used.

The treatment effect was expressed as the difference in change from baseline in FEV1 at 24 weeks between UMEC/VI and tiotropium. FEV1 was selected as it was the primary endpoint in UMEC/VI clinical trial programme. The treatment effect was estimated by a random-effects meta-analysis of tiotropium comparator trials from the UMEC/VI phase 3a clinical programme (DB2113360, DB2113374, ZEP117115) [13, 14]. Trials results demonstrated that UMEC/VI was superior to tiotropium on FEV1 indicating that it provides superior bronchodilation to tiotropium. The treatment benefit of UMEC/VI over tiotropium was assumed for the lifetime of the patient in the base case. A separate scenario analysis was conducted (Scenario C) assuming that the treatment effect lasts for a period of 12 months from treatment initiation, based on the results of UMEC/VI safety study (DB2113359) [23].

### Costs

The perspective adopted for costs was that of the National Health Service (NHS) in England and Wales. The reference year used for costs was 2011–2012. Productivity losses, although significant, were omitted because of this choice of perspective.

Cost of treatment with tiotropium was obtained from the British National Formulary (BNF March 2014) and was estimated to be £33.50 for a 30-day supply [24]. The cost of UMEC/VI was assumed to be equivalent to tiotropium in the base case and was increased to be equivalent to the price of indacaterol plus tiotropium in separate inhalers (£62.76) in the sensitivity analyses. Resource-use costs were estimated using NHS reference costs for 2013–2014 and are displayed in Table 1.

**Table 1:** Resource use costs estimated using NHS reference costs for 2011–2012

Resource use	Costs	Source
Cost per day in ICU	£1190.29	NHS National Schedule of Reference Costs 2013–2014
Cost per day in general ward	£514.00	NHS National Schedule of Reference Costs 2013–2014
Per hospitalisation (COPD Related)	£1897.00	NHS National Schedule of Reference Costs 2013–2014
ER visit	£123.74	NHS National Schedule of Reference Costs 2013–2014
Hospital outpatient visit	£150.00	NHS National Schedule of Reference Costs 2013–2014
Physician visits	Cost (2012£)	
Daytime home visit	£114.00	Personal Social Service Research Unit—Unit Costs of Health and Social Care 2012
Night-time home visit	£114.00	Assumed the same as daytime visit
Visit to physician’s office	£67.00	Personal Social Service Research Unit—Unit Costs of Health and Social Care 2012

*ER* emergency room, *COPD* chronic obstructive pulmonary disease, *ICU* intensive care unit, *NHS* National Health Service

### Cost-effectiveness analyses

The results of the cost-effectiveness analysis are reported here in the form of incremental cost per quality-adjusted life year (QALY) gained. Costs and outcomes were calculated separately for each treatment alternative over the model timeframe and then discounted at 3.5 % per annum [25]. Uncertainty around patient level inputs (first order) and risk-equation estimates (second order) was further explored using probabilistic sensitivity analysis with 5000 iterations. The range of values and the distributions used in the probabilistic sensitivity analyses are presented in Table 2 for patient level inputs and in Additional file 1 for risk equation estimates.

**Table 2:** Model baseline population parameters

Parameter	Values and % of patients	Standard error^a^	Distributions used in the PSA
Gender			
Female (%)	32.0 %	1.15 %	Beta
Mean age (years)	63.3	0.1	Normal
BMI			
Low (%)	10.4 %	0.84 %	Beta
Medium (%)	65.1 %	–	–
High (%)	24.5 %	1.02 %	Beta
Any CVD comorbidity (%)	43.5 %	1.13 %	Beta
Without comorbidity (%)	56.5 %	–	–
Any other comorbidity (%)	77.3 %	1.07 %	Beta
History of exacerbation, 1 or more (%)	46.2 %	1.21 %	Beta
mMRC score ≥2 (%)	100.0 %		–
Current smokers (%)	49.0 %	1.16 %	Beta
Height (cm)	169.0	0.1	Normal
Fibrinogen (µg/mL)	458.8	102.4	Gamma
Number of exacerbations in prior year	0.50	0.01	Gamma
Proportion of prior exacerbations that are severe	20.0		Gamma
Starting SGRQ score	49.1	0.5	Normal
Starting FEV1 %p (%)	47.7 %	0.2 %	Beta
6-min walk distance (m)	378.3	2.9	Normal

*BMI* body mass index, *CVD* cardiovascular disease, *ECLIPSE* Evaluation of COPD Longitudinally to Identify Predictive Surrogate Endpoints, *FEV1%p* forced expiratory volume in 1 s percent predicted, *mMRC* modified Medical Research Council dyspnoea scale, *PSA* probabilistic sensitivity analysis, *SE* standard error, *SGRQ* St. George’s Respiratory Questionnaire

^a^ *SE* Calculated or assumed based on availability of data

## Results

### Baseline cohort

The baseline demographic and clinical parameters used in the model are displayed in Table 2. Cohort characteristics assumed that all patients were symptomatic (modified Medical Research Council scale [mMRC] score ≥2) and broadly comparable to symptomatic patients in the primary care setting in the United Kingdom [26].

### UMEC/VI treatment effect

**Fig. 2:**
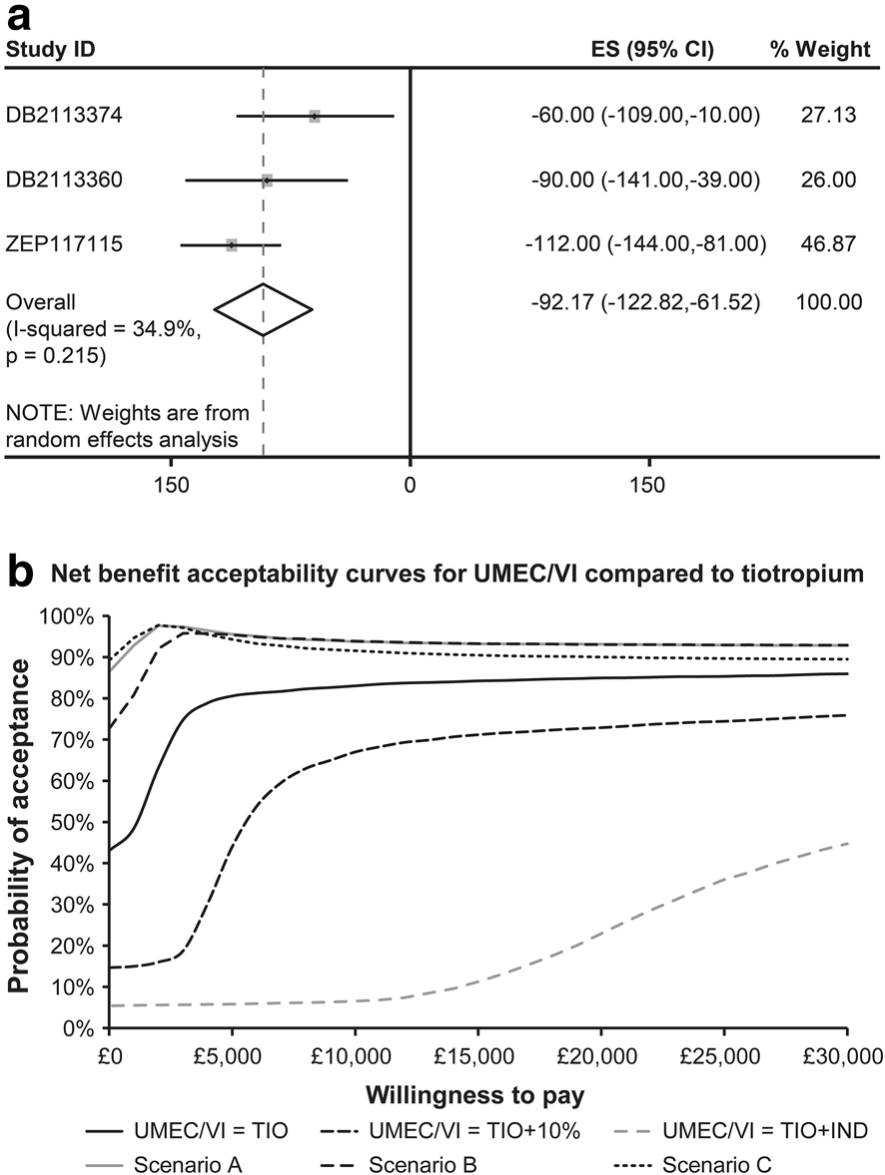
**a** Random-effects meta-analysis of tiotropium comparator trials. **b** Probabilistic sensitivity analysis: net benefit acceptability curves for UMEC/VI compared with tiotropium. *CI* confidence interval, *ES* effect size, *IND* indacaterol, *TIO* tiotropium, *UMEC/VI* umeclidinium bromide/vilanterol. DB2113374 and DB2113360 [11]; ZEP117115 [12]. **a** Effect size (ES) represents increment in FEV1 (ml) of UMEC/VI compared to tiotropium. **b** Each *line* on the *graph* represents probability of acceptance of UMEC/VI compared to tiotropium under a particular scenario at multiple thresholds of willingness to pay

The random-effects meta-analysis of tiotropium comparator trials estimated the treatment effect of UMEC/VI to be 92.17 (maximum likelihood; 95 % confidence interval: 61.52, 122.82; Fig. 2a) [13, 14].

### Model predictions

In the base case, UMEC/VI resulted in 5.35 moderate exacerbations and 4.32 severe exacerbations per patient over patient lifetime. The corresponding predictions for tiotropium were 5.35 and 4.30, respectively. The FEV1 benefit of UMEC/VI resulted in an estimated annual exacerbation reduction of 0.04 exacerbations per patient. UMEC/VI also resulted in 0.36 incremental life years and 0.18 incremental QALYs compared with tiotropium over patient lifetime (Table 3).

**Table 3:** Cost effectiveness of UMEC/VI compared with tiotropium

Scenario	Incremental QALYs	Incremental costs	ICER
Base case	0.18	£372.29	£2087.60
UMEC/VI price = tiotropium + 10 %	0.18	£687.81	£3856.87
UMEC/VI price = tiotropium + indacaterol	0.18	£3128.15	£17,540.98
Scenario A	0.01	−£0.89	Dominant
Scenario B	0.04	£22.69	£567.04
Scenario C	0.01	−£3.67	Dominant

*ICER* incremental cost-effectiveness ratio, *QALY* quality-adjusted life year, *UMEC/VI* umeclidinium bromide-vilanterol

In the base case, the treatment and resource use costs were broadly comparable between the two treatment alternatives. Per-patient costs over patient lifetime were £372.29 more for UMEC/VI compared to tiotropium. The resultant incremental cost-effectiveness ratio (ICER) was £2087.60 per QALY. This ICER increased to £3856.87/QALY and £17,540.98/QALY with increase in UMEC/VI price to £36.85 (10 % premium to tiotropium) and £62.76 (equivalent to price of indacaterol plus tiotropium in separate inhalers), respectively. At a willingness to pay of £20,000 per QALY, the probability of UMEC/VI being cost-effective was 84.9 %. This changed to 72.9 % and 22.9 % as the price of UMEC/VI increased to £36.85 and £62.76, respectively (Fig. 2b).

Reducing the model timeframe to 1 year (Scenario A) and 5 years (Scenario B) resulted in improvements in ICER for UMEC/VI. In Scenario A, UMEC/VI resulted in 0.04 fewer exacerbations and 0.01 incremental QALYs compared to tiotropium. UMEC/VI also resulted in lower annual treatment cost of £0.89, thereby dominating tiotropium. In Scenario B, UMEC/VI predicted 0.04 fewer exacerbations per year, 0.04 incremental QALYs, and an ICER of £567.04 per QALY. UMEC/VI in Scenario C, which assumed treatment benefit for a period of 12 months from treatment initiation, resulted in 0.01 fewer exacerbations, 0.01 incremental QALYs and £3.67 lower costs resulting in UMEC/VI dominating tiotropium. At a willingness to pay threshold of £20,000 per QALY, the probability of UMEC/VI being cost-effective was 93.1, 93.0 and 90.0 % for Scenarios A, Scenario B and Scenario C, respectively.

## Discussion

Economic assessments in COPD have demonstrated combination therapies to be cost-effective in the past, but the results have often been inconsistent and depend on the choice of products in the combination and the comparators. Friedman et al. reported that the combination of albuterol and ipratropium is associated with lower rates of exacerbations and is more cost-effective than either drug as monotherapy [27]. Similarly, fluticasone and salmeterol combination therapy was found to be cost-effective in comparison to ipratropium alone [28], ipratropium/albuterol (IPA), and tiotropium alone [29]. However, the combination of tiotropium and salmeterol was not an economically attractive alternative to tiotropium monotherapy [30]. Therefore, it was important to assess the cost-effectiveness of UMEC/VI, a new combination bronchodilator compared with tiotropium, which is the current standard of care in symptomatic COPD patients.

UMEC/VI has been shown to be an efficacious treatment compared with tiotropium [13, 14]. This study assessed the cost-effectiveness of UMEC/VI versus tiotropium monotherapy by using a treatment-specific COPD economic model. The model was based on robust long-term multicentre studies in COPD such as the ECLIPSE [19] and Towards a Revolution in COPD Health (TORCH) [31] and was further validated using Understanding Potential Long-term Impacts on Function with Tiotropium (UPLIFT) study results [32]. In addition, extensive internal and external validation was undertaken to assess its suitability to COPD patient populations likely to receive a combination bronchodilator such as UMEC/VI in clinical practice. Overall, the model demonstrated acceptable content and predictive validity.

The model framework allowed FEV1 benefit observed in the clinical trial programme for UMEC/VI to predict long-term outcomes such as exacerbations, mortality and health status. Results suggested small improvements in exacerbation rates and QALYs for UMEC/VI compared with tiotropium. This is not surprising when considering that the patient cohort had low risk of exacerbations with moderate disease severity and dyspnoea. This coupled with low to medium correlation between FEV1 and patient-reported outcomes may not have allowed treatment effect to be completely translated into patient-reported outcomes. The model allows treatment effect to be applied based on SGRQ and to test this hypothesis we used results from one of the UMEC/VI studies where UMEC/VI demonstrated significant benefit compared with tiotropium [14]. The model predicted higher QALY benefit (0.35 vs 0.18) and, therefore, more favourable outcome for UMEC/VI when treatment effect on SGRQ was used instead of FEV1. However, we did not use these results in our base case as SGRQ was not the primary endpoint in UMEC/VI clinical studies and UMEC/VI benefit over tiotropium was not always significant [13, 14].

A key assumption in the model was the duration of the treatment effect. In the base case, we assumed the treatment effect to continue over the lifetime of the patient. This is in line with the other published COPD models in literature [33]. A scenario analysis assuming a shorter duration of treatment effect of 12 months was also performed. This choice of duration was based on UMEC/VI safety study [23], which demonstrated the benefit of UMEC/VI on lung function up to 12 months after treatment initiation. Results showed that UMEC/VI ICER improved with a shorter treatment effect than when the treatment effect lasted over patient lifetime. This counterintuitive finding may be a result of patients in UMEC/VI treatment arm living longer and, thereby, incurring higher costs later in their life.

### Limitations

COPD is a chronic progressive condition, and patients with COPD frequently undergo treatment switches or escalations. In the current analysis, we did not consider any treatment changes. A combination therapy such as UMEC/VI is a new class of combination bronchodilators currently being introduced, and its impact on the treatment pathway is yet unknown. In addition, we assumed that any changes to patient therapy will be similar in UMEC/VI and tiotropium treatment arms such that there will be no additional benefit of UMEC/VI in delaying treatment escalation. We believe that this is a conservative assumption and unlikely to significantly impact final conclusions.

## Conclusion

Overall, UMEC/VI can be considered a cost-effective alternative to tiotropium. Further evidence on UMEC/VI is needed to assess its long-term benefit for COPD patients. Along with other dual bronchodilators, this may provide additional options in the armamentarium to physicians for COPD patients in need of bronchodilator treatment.

## Additional file

10.1186/s12962-015-0048-6 List of risk equations incorporated into the disease progression model (linked-equation model).

## Abbreviations

COPD: chronic obstructive pulmonary disease
DALYs: disability-adjusted life years
EQ-5D: EuroQol 5 dimension
ECLIPSE: Evaluation of COPD Longitudinally to Identify Predictive Surrogate Endpoints
FEV1: forced expiratory volume in 1 s
GOLD: Global Initiative for Chronic Obstructive Lung Disease
ICERs: incremental cost-effectiveness ratios
IPA: ipratropium/albuterol
LAMA: long-acting muscarinic antagonist
LABA: long-acting β2-agonist
6MWT: 6-minute walk test
mMRC: modified Medical Research Council scale
NHS: National Health Services
QALYs: quality-adjusted life years
SGRQ-C: St George’s Respiratory Questionnaire for COPD
TORCH: Towards a Revolution in COPD Health
UMEC/VI: umeclidinium bromide and vilanterol combination
UPLIFT: Understanding Potential Long-term Impacts on Function with Tiotropium
