# Supplementary material for: Cost-effectiveness of umeclidinium/vilanterol combination therapy compared to tiotropium monotherapy among symptomatic patients with chronic obstructive pulmonary disease in the UK
Source: Cost Eff Resour Alloc. 2015 Dec 12;13:22. doi: 10.1186/s12962-015-0048-6 (PMC4676898; doi:10.1186/s12962-015-0048-6)
Supplement: Supplementary file 1 — 10.1186/s12962-015-0048-6 List of risk equations incorporated into the disease progression model (linked-equation model). [file 12962_2015_48_MOESM1_ESM.docx]

**Additional file 1**

**Title: Cost-Effectiveness of Umeclidinium/Vilanterol Combination Therapy Compared To Tiotropium Monotherapy Among Symptomatic Patients With Chronic Obstructive Pulmonary Disease in the UK**

**Table S1 - List of risk equations incorporated into the disease progression model (linked-equation model)**

|  | **RE1a: moderate exacerbations** | | | **RE1b: severe exacerbations** | | | **RE2: FEV1** | | | **RE3A1: symptoms, dyspnoea, level 2** | | |
| --- | --- | --- | --- | --- | --- | --- | --- | --- | --- | --- | --- | --- |
|  |  | **95% CI** | |  | **95% CI** | |  | **95% CI** | |  | **95% CI** | |
| **Explanatory variable** | **Effect** | **Lower** | **Upper** | **Effect** | **Lower** | **Upper** | **Effect** | **Lower** | **Upper** | **Effect** | **Lower** | **Upper** |
| Intercept | -1.1842 | -1.87 | -0.50 | -3.87 | -5.35 | -2.39 | -1031.09 | -1593.35 | -468.82 | 1.6232 | -0.7831 | 4.0295 |
| Time (years) | -0.0729 | -0.1164 | -0.0293 | 0.1604 | 0.0663 | 0.2545 | -26.7231 | -32.9069 | -20.5394 | 0.0000 | -0.1855 | 0.1856 |
| FEV1 %predicted_t-1_ | -0.0082 | -0.0113 | -0.0050 | -0.0270 | -0.0354 | -0.0186 | - | - | - | 0.0376 | 0.0252 | 0.0500 |
| Dysp_t-1_ (several vs none) | 0.0858 | -0.0524 | 0.2240 | 0.0849 | -0.2428 | 0.4126 | - | - | - | - | - | - |
| Dysp_t-1_ (most days vs. none) | 0.1394 | -0.0009 | 0.2797 | 0.1098 | -0.2319 | 0.4514 | - | - | - | - | - | - |
| Cough&Sput_t-1_ | 0.0471 | -0.0304 | 0.1247 | 0.1057 | -0.0560 | 0.2675 | - | - | - | - | - | - |
| MExac_t-1 to t_ | - | - | - | - | - | - | -7.9655 | -12.4854 | -3.4457 | - | - | - |
| SExac_t-1 to t_ | - | - | - | - | - | - | -9.6954 | -18.0287 | -1.3620 | - | - | - |
| Height_(0)_ | - | - | - | - | - | - | 19.7857 | 17.0234 | 22.5480 | - | - | - |
| Age_(0)_ | 0.0016 | -0.0064 | 0.0096 | 0.0191 | 0.0013 | 0.0370 | -9.8944 | -12.8081 | -6.9806 | -0.0347 | -0.0620 | -0.0074 |
| Sex_(0)_ (female vs. male) | 0.2308 | 0.1173 | 0.3443 | 0.0538 | -0.1661 | 0.2736 | -78.2544 | -131.0900 | -25.4222 | -0.1224 | -0.5141 | 0.2692 |
| 6MWT_(0)_ (m) | 0.0005 | -0.0001 | 0.0010 | -0.0002 | -0.0012 | 0.0009 | 0.6187 | 0.4290 | 0.8084 | -0.0015 | -0.0034 | 0.0003 |
| BMI_(0)_ (low vs normal) | -0.1243 | -0.2878 | 0.0393 | 0.2620 | -0.0058 | 0.5298 | -163.6700 | -220.3300 | -107.0100 | -0.4242 | -1.0148 | 0.1663 |
| BMI_(0)_ (high vs. normal) | -0.1047 | -0.2397 | 0.0304 | -0.0612 | -0.3292 | 0.2067 | 171.7500 | 124.0900 | 219.4100 | -0.1566 | -0.6166 | 0.3035 |
| CVD comorbidity_(0)_ (yes vs. no) | -0.1324 | -0.2544 | -0.0103 | 0.1858 | -0.0414 | 0.4129 | 47.0216 | 5.3665 | 88.6768 | 0.0400 | -0.3645 | 0.4445 |
| Other comorbidity_(0)_ (yes vs. no) | 0.1102 | -0.0165 | 0.2369 | 0.0077 | -0.2376 | 0.2529 | 97.0406 | 52.5480 | 141.5300 | -0.2902 | -0.7050 | 0.1246 |
| Current smoker_(0)_ | -0.1412 | -0.2654 | -0.0169 | 0.0625 | -0.1723 | 0.2972 | 116.0900 | 69.4793 | 162.7000 | -0.1640 | -0.5551 | 0.2271 |
| Fibrinogen_(0)_ (mg/dl) | 0.0008 | 0.0003 | 0.0013 | 0.0010 | 0.0000 | 0.0019 | -0.7307 | -0.9230 | -0.5384 | -0.0012 | -0.0032 | 0.0007 |
| mMRC ≥ 2_(0)_ | 0.0271 | -0.0982 | 0.1524 | -0.0651 | -0.3429 | 0.2127 | -112.2600 | -158.6000 | -65.9184 | -0.0800 | -0.5297 | 0.3696 |
| SGRQ-C_(0)_ | 0.0062 | 0.0029 | 0.0095 | 0.0191 | 0.0116 | 0.0266 | -4.2412 | -5.4490 | -3.0334 | -0.0673 | -0.0801 | -0.0546 |
| Prior exacerbations_(0)_ (one or more vs. zero) | 0.8276 | 0.7158 | 0.9393 | 0.5580 | 0.3427 | 0.7733 | -94.2009 | -133.5700 | -54.8316 | -0.1422 | -0.5201 | 0.2356 |

**Table 1(continued) - List of risk equations incorporated into the disease progression model (linked-equation model)**

|  | **RE3A2: symptoms, dyspnoea, level 3** | | | **RE3B: cough and sputum** | | | **RE4: exercise capacity (6MWT distance)** | | |
| --- | --- | --- | --- | --- | --- | --- | --- | --- | --- |
|  |  | **95% CI** | |  | **95% CI** | |  | **95% CI** | |
| **Explanatory variable** | **Effect** | **Lower** | **Upper** | **Effect** | **Lower** | **Upper** | **Effect** | **Lower** | **Upper** |
| Intercept | -2.5711 | -4.312 | -0.83 | -4.1958 | -6.056 | -2.336 | 733.67 | 679.05 | 788.3 |
| Time (years) | 0.03873 | -0.091 | 0.168 | -0.0407 | -0.168 | 0.0869 | -7.8837 | -11.0384 | -4.729 |
| FEV1 %predicted_t-1_ | -0.0456 | -0.055 | -0.036 | -0.0062 | -0.016 | 0.0032 | 1.4729 | 1.1707 | 1.7752 |
| Dysp_t-1_ (several vs none) | - | - | - | - | - | - | 6.1632 | -3.9464 | 16.2728 |
| Dysp_t-1_ (most days vs. none) | - | - | - | - | - | - | 0.5775 | -10.2914 | 11.4463 |
| Cough&Sput_t-1_ | - | - | - | - | - | - | 1.0111 | -5.7966 | 7.8188 |
| MExac_t-1 to t_ | - | - | - | - | - | - | -1.2831 | -3.9059 | 1.3396 |
| SExac_t-1 to t_ | - | - | - | - | - | - | -13.376 | -18.6102 | -8.1419 |
| height_(0)_ | - | - | - | - | - | - | - | - | - |
| Age_(0)_ | 0.02727 | 0.0073 | 0.0473 | 0.0228 | 0.0014 | 0.0442 | -3.834 | -4.5538 | -3.1143 |
| Sex_(0)_ (female vs. male) | 0.2147 | -0.071 | 0.5006 | -0.6526 | -0.962 | -0.344 | -27.3677 | -37.7622 | -16.9733 |
| 6MWT_(0)_ (m) | -0.0004 | -0.002 | 0.0009 | -0.0001 | -0.002 | 0.0013 | - | - | - |
| BMI_(0)_ (low vs normal) | -0.0097 | -0.402 | 0.3829 | 0.1112 | -0.307 | 0.529 | -14.1001 | -28.638 | 0.4379 |
| BMI_(0)_ (high vs. normal) | 0.2594 | -0.07 | 0.5891 | -0.391 | -0.747 | -0.035 | -41.1978 | -53.1039 | -29.2917 |
| CVD comorbidity_(0)_ (yes vs. no) | -0.1517 | -0.439 | 0.1352 | -0.5048 | -0.815 | -0.194 | -13.3929 | -23.9583 | -2.8274 |
| Other comorbidity_(0)_ (yes vs. no) | 0.3211 | 0.0133 | 0.6289 | 0.4121 | 0.0808 | 0.7434 | -1.5096 | -12.734 | 9.7148 |
| Current smoker_(0)_ | -0.0503 | -0.334 | 0.2332 | 1.7279 | 1.3973 | 2.0586 | -12.48 | -23.0063 | -1.9538 |
| Fibrinogen_(0)_ (mg/dl) | 0.0007 | -7E-04 | 0.0021 | 0.00127 | -2E-04 | 0.0027 | -0.08636 | -0.1349 | -0.03777 |
| mMRC ≥ 2_(0)_ | 0.1445 | -0.167 | 0.4561 | 0.00483 | -0.334 | 0.344 | -46.4062 | -57.8632 | -34.9491 |
| SGRQ-C_(0)_ | 0.05802 | 0.0486 | 0.0674 | 0.03523 | 0.026 | 0.0445 | -1.5663 | -1.8718 | -1.2608 |
| Prior exacerbations_(0)_ (one or more vs. zero) | 0.0721 | -0.198 | 0.3417 | 0.4048 | 0.1154 | 0.6941 | -12.303 | -22.4341 | -2.1718 |

**Table 1 (continued) - List of risk equations incorporated into the disease progression model (linked-equation model)**

|  | **FO1: SGRQ-C score** | | | **FO2: mortality, (SAS PHREG Model; Cox Model)** | | | **FO2: mortality, Weibull (SAS LIFEREG Model)** | | |
| --- | --- | --- | --- | --- | --- | --- | --- | --- | --- |
|  |  | **95% CI** | |  | **95% CI** | |  | **95% CI** | |
| **Explanatory variable** | **Effect** | **Lower** | **Upper** | **Effect** | **Lower** | **Upper** | **Effect** | **Lower** | **Upper** |
| Intercept | 71.1550 | 63.4624 | 78.8477 | - | - | - | 10.9512 | 9.2599 | 12.6424 |
| Time (years) | 0.6125 | 0.1872 | 1.0377 | - | - | - | - | - | - |
| MExac_t-1 to t_ | 0.8524 | 0.5153 | 1.1894 | 0.0487 | 0.0615 | 1.0500 | -0.0605 | -0.1531 | 0.0322 |
| SExac_t-1 to t_ | 1.9092 | 1.2779 | 2.5406 | 0.1603 | 0.0962 | 1.1740 | -0.0528 | -0.2608 | 0.1551 |
| FEV_1_ %predicted_t_ | -0.0060 | -0.0072 | -0.0047 | -0.0126 | 0.0060 | 0.9870 | 0.0103 | 0.0011 | 0.0194 |
| Dysp_t-1_ (Several vs none) | 9.6256 | 8.2969 | 10.9543 | -0.0990 | 0.3174 | 0.9060 | 0.0807 | -0.3949 | 0.5562 |
| Dysp_t-1_ (most days vs. none) | 17.5914 | 16.1742 | 19.0086 | 0.0307 | 0.3151 | 1.0310 | -0.0442 | -0.5171 | 0.4287 |
| Cough&Sput_t_ | 5.3077 | 4.4332 | 6.1823 | -0.0717 | 0.1596 | 0.9310 | 0.0815 | -0.1583 | 0.3213 |
| 6MWT_t_ | -0.0266 | -0.0318 | -0.0214 | -0.0034 | 0.0008 | 0.9970 | 0.0024 | 0.0012 | 0.0037 |
| Age_(0)_ | -0.3725 | -0.4611 | -0.2838 | 0.0526 | 0.0125 | 1.0540 | -0.0419 | -0.0615 | -0.0223 |
| Sex_(0)_ (female vs. male) | -4.3177 | -5.6265 | -3.0088 | -0.2383 | 0.1715 | 0.7880 | 0.1673 | -0.0903 | 0.4249 |
| 6MWT_(0)_ (m) | -0.0153 | -0.0218 | -0.0088 | - | - | - | - | - | - |
| BMI_(0)_ (low vs normal) | 1.4148 | -0.3330 | 3.1627 | 0.4434 | 0.1993 | 1.5580 | -0.3337 | -0.6355 | -0.0319 |
| BMI_(0)_ (high vs. normal) | 0.5468 | -0.9250 | 2.0187 | 0.0239 | 0.1867 | 1.0240 | -0.0583 | -0.3374 | 0.2207 |
| CVD comorbidity_(0)_ (yes vs. no) | 1.7147 | 0.4382 | 2.9912 | 0.3716 | 0.1525 | 1.4500 | -0.2590 | -0.4915 | -0.0264 |
| Other comorbidity_(0)_ (yes vs. no) | 1.6157 | 0.2566 | 2.9747 | 0.0945 | 0.1827 | 1.0990 | -0.0813 | -0.3578 | 0.1952 |
| Current smoker_(0)_ | 0.0702 | -1.2110 | 1.3513 | 0.2139 | 0.1643 | 1.2390 | -0.1725 | -0.4189 | 0.0739 |
| Fibrinogen_(0)_ (mg/dl) | -0.0009 | -0.0068 | 0.0050 | 0.0017 | 0.0006 | 1.0020 | -0.0014 | -0.0024 | -0.0005 |
| mMRC ≥2_(0)_ | 9.6749 | 8.3594 | 10.9903 | 0.0366 | 0.1899 | 1.0370 | -0.0030 | -0.2898 | 0.2839 |
| SGRQ-C_(0)_ | - | - | - | -0.0012 | 0.0054 | 0.9990 | 0.0003 | -0.0078 | 0.0083 |
| Prior exacerbations_(0)_ (one or more vs. zero) | 2.7953 | 1.5652 | 4.0254 | - | - | - | - | - | - |
| Scale | - | - | - | - | - | - | 0.7624 | 0.6648 | 0.8744 |
| Weibull Shape | - | - | - | - | - | - | 1.3116 | 1.1437 | 1.5042 |

**Table 1 (continued) - List of risk equations incorporated into the disease progression model (linked-equation model)**

|  | **FO3A: resource use, general ward days** | | | **FO3B: resource use, ICU days** | | | **FO3C: resource use, ER visits** | | | **FO3D: resource use, office visits** | | |
| --- | --- | --- | --- | --- | --- | --- | --- | --- | --- | --- | --- | --- |
|  |  | **95% CI** | |  | **95% CI** | |  | **95% CI** | |  | **95% CI** | |
| **Explanatory variable** | **Effect** | **Lower** | **Upper** | **Effect** | **Lower** | **Upper** | **Effect** | **Lower** | **Upper** | **Effect** | **Lower** | **Upper** |
| Intercept | -1.0688 | -1.7276 | -0.4101 | -2.1133 | -4.2101 | -0.0166 | -2.3762 | -4.2039 | -0.5485 | -0.3691 | -1.1591 | 0.4209 |
| Time (years) | 0.1103 | 0.0369 | 0.1836 | 0.5465 | 0.2952 | 0.7979 | -0.0870 | -0.2969 | 0.1229 | 0.0531 | -0.0369 | 0.1430 |
| MExac_t-1 to t_ | -0.0781 | -0.0939 | -0.0624 | 0.0245 | -0.0161 | 0.0650 | 0.0760 | 0.0427 | 0.1093 | 0.1105 | 0.0981 | 0.1230 |
| SExac_t-1 to t_ | 0.4721 | 0.4518 | 0.4925 | 0.4223 | 0.3608 | 0.4838 | 0.2387 | 0.1736 | 0.3038 | 0.0391 | -0.0129 | 0.0912 |
| FEV_1_ %predicted_t_ | -0.0149 | -0.0190 | -0.0108 | -0.0238 | -0.0377 | -0.0100 | -0.0200 | -0.0319 | -0.0082 | -0.0108 | -0.0158 | -0.0058 |
| Dysp_t-1_ (Several vs none) | 0.2679 | 0.0388 | 0.4969 | -0.0624 | -0.6960 | 0.5712 | 0.1653 | -0.4980 | 0.8286 | 0.4651 | 0.1161 | 0.8142 |
| Dysp_t-1_ (most days vs. none) | 0.2125 | -0.0232 | 0.4482 | -0.5286 | -1.2178 | 0.1607 | 0.1244 | -0.5732 | 0.8219 | 0.6740 | 0.3144 | 1.0335 |
| Cough&Sput_t_ | 0.1864 | 0.0660 | 0.3067 | -0.0344 | -0.4437 | 0.3750 | -0.0627 | -0.4055 | 0.2801 | 0.1559 | 0.0074 | 0.3044 |
| Age_(0)_ | 0.0105 | 0.0032 | 0.0178 | -0.0029 | -0.0275 | 0.0216 | 0.0157 | -0.0056 | 0.0370 | -0.0114 | -0.0201 | -0.0027 |
| Sex_(0)_ | -0.1495 | -0.2951 | -0.0039 | -0.9816 | -1.5658 | -0.3974 | -0.4055 | -0.8142 | 0.0032 | 0.1847 | 0.0287 | 0.3407 |
| BMI (low vs normal)_(0)_ | 0.2960 | 0.1678 | 0.4241 | 0.2310 | -0.2199 | 0.6819 | 0.3220 | -0.0449 | 0.6889 | -0.2544 | -0.4428 | -0.0661 |
| BMI (high vs. normal)_(0)_ | -0.3781 | -0.5557 | -0.2005 | 0.0403 | -0.4761 | 0.5567 | -0.6200 | -1.1691 | -0.0709 | -0.2485 | -0.4379 | -0.0591 |
| CVD comorbidity_(0)_ | 0.0269 | -0.0996 | 0.1535 | 0.6546 | 0.2688 | 1.0404 | -0.1036 | -0.4877 | 0.2806 | -0.0645 | -0.2312 | 0.1022 |
| Other comorbidity_(0)_ | -0.1690 | -0.3047 | -0.0334 | 0.3295 | -0.1954 | 0.8544 | -0.2442 | -0.6403 | 0.1520 | 0.0262 | -0.1691 | 0.2214 |
| Current smoker_(0)_ | -0.1992 | -0.3185 | -0.0799 | 0.3026 | -0.0922 | 0.6974 | -0.1766 | -0.5235 | 0.1703 | -0.2163 | -0.3653 | -0.0673 |
| mMRC_(0)_ | -0.0046 | -0.2553 | 0.2461 | -0.7611 | -1.3680 | -0.1543 | -0.4203 | -0.9795 | 0.1390 | -0.1588 | -0.4185 | 0.1009 |
| SGRQ_(0)_ | 0.0147 | 0.0109 | 0.0185 | 0.0142 | 0.0015 | 0.0268 | 0.0046 | -0.0061 | 0.0152 | -0.0016 | -0.0063 | 0.0030 |
| Prior exacerbations_0_ (lvl2 vs. lvl1) | 0.4613 | 0.3383 | 0.5843 | -0.0076 | -0.3889 | 0.3737 | 0.2226 | -0.1104 | 0.5557 | 0.3351 | 0.1898 | 0.4805 |

**Table 1 (continued) - List of risk equations incorporated into the disease progression model (linked-equation model)**

|  | **FO3E: resource use, day/night home visits** | | | **FO3F: resource use, outpatient visit days** | | | **FO3G: resource use, proportion hospitalized** | | |
| --- | --- | --- | --- | --- | --- | --- | --- | --- | --- |
|  |  | **95% CI** | |  | **95% CI** | |  | **95% CI** | |
| **Explanatory variable** | **Effect** | **Lower** | **Upper** | **Effect** | **Lower** | **Upper** | **Effect** | **Lower** | **Upper** |
| Intercept | -7.1905 | -10.5055 | -3.8755 | -0.9087 | -2.1694 | 0.3519 | -2.7609 | -3.6546 | -1.8673 |
| Time (years) | -0.3722 | -0.7330 | -0.0114 | -0.1898 | -0.3425 | -0.0371 | -0.0004 | 0.0530 | 0.0001 |
| MExac_t-1 to t_ | 0.0243 | -0.0452 | 0.0938 | 0.0844 | 0.0589 | 0.1100 | -0.0288 | 0.0126 | 5.2181 |
| SExac_t-1 to t_ | 0.1544 | -0.0022 | 0.3110 | 0.0406 | -0.0485 | 0.1298 | 2.0241 | 0.0495 | 1673.7464 |
| FEV_1_ %predicted_t_ | -0.0106 | -0.0291 | 0.0079 | -0.0123 | -0.0202 | -0.0044 | -0.0273 | 0.0029 | 88.9716 |
| Dysp_t-1_ (several vs none) | -0.3437 | -1.1905 | 0.5031 | -0.1533 | -0.5544 | 0.2479 | -0.0061 | 0.0641 | 0.0092 |
| Dysp_t-1_ (most days vs. none) | -0.8936 | -1.8308 | 0.0435 | -0.5760 | -1.0130 | -0.1390 | -0.1144 | 0.0705 | 2.6353 |
| Cough&Sput_t_ | -0.0562 | -0.6063 | 0.4940 | 0.1369 | -0.1030 | 0.3768 | 0.0328 | 0.0864 | 0.1442 |
| Age_(0)_ | 0.0297 | -0.0053 | 0.0648 | -0.0011 | -0.0156 | 0.0134 | 0.0023 | 0.0052 | 0.1947 |
| Sex_(0)_ | -0.1454 | -0.7972 | 0.5063 | -0.6256 | -0.9357 | -0.3155 | -0.1773 | 0.0499 | 12.6120 |
| BMI (low vs normal)_(0)_ | 0.2669 | -0.3350 | 0.8687 | 0.2183 | -0.0504 | 0.4869 | 0.2869 | 0.0694 | 17.1035 |
| BMI (high vs. normal)_(0)_ | -0.7955 | -1.7509 | 0.1599 | -0.6665 | -1.0561 | -0.2768 | -0.3331 | 0.0793 | 17.6573 |
| CVD comorbidity_(0)_ | 0.1027 | -0.4805 | 0.6859 | 0.0388 | -0.2226 | 0.3003 | -0.1006 | 0.0936 | 1.1557 |
| Other comorbidity_(0)_ | 0.3729 | -0.3412 | 1.0869 | -0.2584 | -0.5279 | 0.0110 | -0.1160 | 0.0997 | 1.3555 |
| Current smoker_(0)_ | -0.1645 | -0.7229 | 0.3938 | -0.4398 | -0.6877 | -0.1918 | -0.1536 | 0.0861 | 3.1800 |
| mMRC_(0)_ | 0.9875 | -0.6672 | 2.6422 | 0.0975 | -0.3818 | 0.5769 | 0.1094 | 0.1766 | 0.3836 |
| SGRQ_(0)_ | 0.0127 | -0.0042 | 0.0296 | 0.0140 | 0.0065 | 0.0214 | 0.0128 | 0.0027 | 23.2356 |
| Prior exacerbations_0_ (lvl2 vs. lvl1) | 1.3104 | 0.6188 | 2.0019 | 0.4280 | 0.1844 | 0.6717 | 0.4775 | 0.0847 | 31.8176 |

6MWT, 6-minute walk test; FEV1, forced expiratory volume in 1 second; SGRQ-C, St. George’s Respiratory Questionnaire for chronic obstructive pulmonary disease.

The risk equations were derived from analysis using data from the ECLIPSE study (2,164 patients) for clinical endpoints (RE1-RE4 and FO1-FO2) and the TORCH study (6,108 patients) for resource use (FO3) [16, 29].
